# Food allergy: the molecular and clinical analysis of soybean

**DOI:** 10.1186/2045-7022-1-S1-O27

**Published:** 2011-08-12

**Authors:** Malvina Petronyte, Ruta Dubakiene, Vaclovas Jurgelevicius

**Affiliations:** 1 Vilnius University, Nature Sciences, Vilnius, Lithuania; 2 Vilnius University, Vilnius, Lithuania; 3 National Food and Veterinary Risk Assessment Institute, Biology and Genetically Modified Organism Unit, Vilnius, Lithuania

## Background

There have not been done any studies to evaluate the impact of GMO on human health in Lithuania yet. The aim of our investigation was to evaluate the gauge of soybean allergy in Lithuania, through molecular methods to estimate the pervasion of GM forms between soy and types of modifications and also to evaluate possible impact of GM soy to allergies.

## Methods

Biotechnological methods: PCR, electrophoresis and real – time PCR was used to find allergenic products that were GM as well and what types of modifications had been done to them.

## Results

Through biotechnological methods such as PCR and electrophoresis there were determined if products, used in our project, were pure, without any intermixture of others products. By using Real – time PCR we found out if our product is genetically modified or not. In our case there were two main modifications 35S promoter and NosT terminator. One of these products is soybean, which were used for further testing. From this type of soy prepared 20% hydrolizates were obtained that have been used to perform skin – prick tests on patients who are allergic to wild – type soy. By doing this clinical testing we were trying to find out if GM products may elicit stronger allergic reaction and to increase allergenicity than wild-type products, in our case soybean. We performed skin prick tests with on 20 patients allergic to soy with wild – type and GM soy, to demonstrate the potential influence of GMO.

## Conclusion

Our data showed that soy is one of the most popular food allergen among Lithuanians. Most common GM among soy was 35S promoter and NosT terminator. There were no significant differences between GM and wild – type soybean allergens of skin – prick testing to patients that are allergic to soybean and its products and also to people that have no any allergic response to wild – type.

